# In Situ Provisioning Wildlife with Food, Water, or Shelter after Bushfires: Using a One Welfare Framework to Guide Responses

**DOI:** 10.3390/ani13223518

**Published:** 2023-11-14

**Authors:** Bidda Jones, Catherine Herbert, Samantha Finnerty, Brooke Kennedy, Amy Lykins, John M. Martin, Phil McManus, David Raubenheimer, Michelle Shaw, Paul D. McGreevy

**Affiliations:** 1 School of Veterinary Science, Faculty of Science, University of Sydney, Sydney, NSW 2006, Australia; bidda.jones@sydney.edu.au; 2 Australian Alliance for Animals, 16 Goodhope Street, Paddington, NSW 2021, Australia; 3 School of Life and Environmental Sciences, University of Sydney, Sydney, NSW 2006, Australia; 4 School of Environmental and Rural Science, University of New England, Armidale, NSW 2353, Australia; 5 School of Psychology, Faculty of Medicine and Health, University of New England, Armidale, NSW 2353, Australia; 6 School of Geosciences, Faculty of Science, University of Sydney, Sydney, NSW 2006, Australia; 7 Charles Perkins Centre, University of Sydney, Sydney, NSW 2006, Australia; 8 Welfare, Conservation and Science, Taronga Conservation Society Australia, Mosman, NSW 2088, Australia

**Keywords:** community motivations, fire, natural disaster, nutrition, water, shelter

## Abstract

**Simple Summary:**

The 2019–2020 Black Summer bushfires had a devastating impact on Australian biodiversity. Many affected human communities felt compelled to intervene by organizing and providing food, water, and/or shelter to affected wildlife in situ (‘wildlife provisioning’). While well intentioned, due to the unprecedented scale and intensity of the fires, a lack of institutional support for wildlife provisioning, and what was revealed to be a lack of scientific evidence, this response was largely uncoordinated and a substantial amount of research is required to determine the beneficial and/or negative outcomes of such practices as a response to habitat destruction by bushfires. We propose a ‘One Welfare’ approach that recognizes the interconnection of human, animal, and environmental welfare and examines the existing literature; local legislation; views of stakeholders; emerging data; and modelling from fire events. There is strong evidence indicating that future bushfire seasons will become longer and more intense in Australia and elsewhere, putting the welfare and survival of millions of wild animals at risk every year. If this approach were implemented, we anticipate that best practice recommendations for stakeholders in different contexts would emerge to determine if, when, and how wildlife provisioning best be conducted, now and into the future.

**Abstract:**

Australia’s 2019–2020 bushfires had a devastating impact on animals, humans, and ecosystems. They also demonstrated the lack of evidence or guidance for wildlife provisioning in response to severe fire events when volunteers and wildlife organisations rose to respond. In addition, the unprecedented scale and intensity of the fires and an absence of institutional support for wildlife provisioning meant that well-intentioned interventions were largely uncoordinated and lacked clear short-term, mid-term, and long-term objectives. Fundamentally, a lack of consensus was revealed on whether any such interventions are advisable. Given the strong evidence indicating that future bushfire seasons will become longer and more intense in Australia and elsewhere, the welfare and survival of millions of wild animals are at risk every year. Understanding the impacts of supplementary resource interventions and contributing to the development of best practice information is crucial to inform the response to the next major fire event. Here, we contextualize the arguments for and against provisioning within a ‘One Welfare’ framework that recognizes that animal welfare, biodiversity, and the environment are intertwined with human welfare and community resilience. We propose that the One Welfare approach can facilitate appropriate consideration of the extant scientific and lay literature; local legislation; views of stakeholders; emerging data; and modelling from historic fire events. As a further step, we see merit in engaging with wildlife provisioners and the broader conservation community to build an evidence base for future wildlife provisioning activities. From an informed position, we can encourage beneficial interventions and reduce the risk of negative outcomes. Finally, we propose controlled experiments (e.g., using hazard reduction burns), ongoing data collection using emergent technology, and longitudinal analysis to address shifting research priorities as the climate changes. We conclude that the ordered collection of the necessary evidence relevant to each of the three stakeholder groups in the One Welfare framework has the greatest potential to support an informed policy platform on wildlife provisioning across Australia that is feasible, legal, and sustainable.

## 1. Introduction

The 2019–2020 Black Summer bushfires had a devastating impact on Australian biodiversity, with estimates of 3 billion animals impacted [1]. While many of those animals died, countless others were taken into care or left in a barren landscape with little to no resources. Many affected human communities felt compelled to intervene by organizing and providing food, water, and/or shelter to affected wildlife in situ (‘wildlife provisioning’), with many such activities being supported by financial donations from the broader Australian and international public. Due to the unprecedented scale and intensity of the fires [2] and a lack of institutional support for wildlife provisioning, this response was largely uncoordinated and ranged from ad hoc activities by volunteers and wildlife organisations [3] to state government-funded aerial food drops targeting threatened species [4]. While well intentioned, the scientific evidence base to inform these actions was revealed to be lacking, and it became clear that substantial research is required to determine the impact of such practices as a response to habitat destruction by bushfires. Clearly, animals may need support after disasters other than fires but their needs under non-fire circumstances are too disparate to merit contemporaneous consideration. 

Humans practice intentional wildlife provisioning in a range of contexts, from recreation to conservation and wildlife management. Backyard bird feeding is practiced by many around the globe [5], while tourism ventures often use provisioning as a mechanism to attract target animals to viewing areas, for example, on bear-watching tours or shark dives [6,7,8]. Similarly, game management often incorporates wildlife provisioning to assist overwintering game species and ensure numbers remain high for the hunting season [9,10]. Unintentional provisioning of wildlife by humans is also well documented, through the addition of new resources to environments associated with agriculture or urbanisation [11]. In each of these contexts, resources are provided to animals in addition to their naturally available resources. However, provisioning is being used much less frequently in a conservation setting, often targeting a specific species that has either lost its natural resources due to anthropogenic influences or requires assistance re-establishing within an area [12,13].

There have been some attempts within the literature to review the costs and benefits of provisioning practices in general [11,14,15,16,17,18], with reported benefits including an increase in weight and reproductive output of provisioned individuals [15,19], and a range of costs from the individual to the ecosystem level, such as an increase in disease transmission [20,21], changes to predator–prey dynamics [22], changes to population dynamics, and shifts in natural species assemblages [23]. However, due to the disproportionate amount of published provisioning studies relating to the provisioning of ‘additional’ resources (i.e., resources provided to animals in addition to their naturally available resources) and the focus of most studies on the provisioning and outcomes for one specific species only, there is still very little understanding of the consequences of wildlife provisioning in the context of acute broad-scale habitat destruction, such as in the aftermath of a severe bushfire. 

Although we include shelter when we consider provisioning in the current commentary, we acknowledge that the data, such as they are, remain heavily weighted towards food and water, and that species vary far more in their motivation to access shelter of a certain size and shape than, say, water. The complexity of interactions when all three types of resources are provided to an array of species adds to the case for a holistic approach.

The ongoing lack of evidence or guidance for wildlife provisioning in response to severe fire events was apparent as volunteers and wildlife organisations rose to action in response to the 2019/2020 fires. While well-intentioned provisioning activities were widespread across Australian communities, they mostly lacked cohesion and were likely based on a range of rapidly changing advice from wildlife organisations. Official advice displayed by the New South Wales National Parks and Wildlife Service (NSW NPWS) prior to Black Summer was that feeding wildlife in situ is not recommended. However, in January 2020, an emergency Food and Water Working Group was formed by Wildlife Health Australia in conjunction with NSW NPWS. This group included both government organisations and wildlife industry experts, to discuss the concerns with provisioning practices being undertaken [24]. While the group identified significant risks involved in supplementary provisioning at the individual, population, and ecosystem levels, the group also developed and published brief guidelines for supplementary food and water during and after the fires. This was deemed necessary as it became clear that (1) wildlife needed assistance from unprecedented habitat destruction, and (2) people were already provisioning, so the development of guidelines was essential to manage these activities and reduce the risk to animals, provisioning personnel, and the environment. These events highlight the complex challenge that wildlife provisioning presents, requiring the integration and management of animal welfare issues and ongoing species conservation, as well as the differing attitudes to wildlife provisioning between policy makers, scientists, and the community.

**Figure 1:**
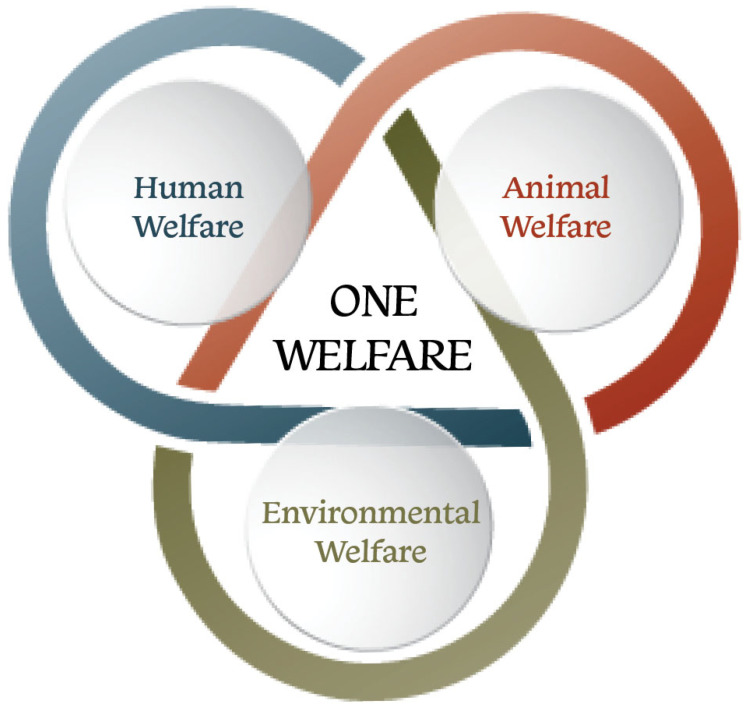
The One Welfare model requires simultaneous consideration of three stakeholder groups: animals, humans, and the environment (with thanks to Cristina Wilkins, *Horses and People* magazine).

As extreme weather events are predicted to increase in frequency (and possibly also severity) due to climate change [25], there is a pressing need to refine our future response to such bushfire emergencies, and specifically, to develop an evidence-based framework for best practice in wildlife provisioning. In this commentary, we propose that wildlife provisioning should be reviewed using a One Welfare approach (See Figure 1) [26,27]—a concept that recognises the interconnections between animal welfare, human welfare, and the environment. For example, potential animal welfare benefits of provisioning bushfire-affected wildlife might include increasing opportunities for food and water and increasing the safety and survival rates of certain wildlife species through ensuring critical levels of hydration, nutrition, and appropriate refuge for affected species [28]. Provisioning may also have environmental benefits, for example, assisting in sparing recovering vegetation from foraging pressures, whilst environmental costs might include changes to natural species assemblages or unintentional spreading of invasive plant seeds in food provisions. Finally, potential benefits to human welfare might include reducing distress and alleviating grief in individuals and communities directly affected by bushfires through the opportunity to take practical steps to assist affected wildlife. Importantly, adopting a One Welfare approach to investigate not only the consequences of provisioning for wildlife welfare and conservation but also the human motivations and attitudes towards provisioning activities, could assist in delivering provisioning guidance for optimal animal welfare outcomes that are also compatible with conservation and acceptable to human stakeholders.

In the following sections, we propose a framework for developing an evidence-based approach to wildlife provisioning after natural disasters using a One Welfare approach. We firstly explore the concept of One Welfare as it relates to wildlife provisioning, and then propose a combination of ecological and social research to assess the consequences of wildlife provisioning in response to natural disasters to determine if, when, and how it should best be conducted.

## 2. One Welfare

The concept of ‘One Welfare’ emphasises the relationships among animal welfare, human welfare, and the environment [26,27]. Although first proposed in the context of veterinary medicine, this concept can be integrated into fields such as environmental and animal welfare policy, sustainability, and conservation to foster interdisciplinary collaboration and improve outcomes for animals, humans, and the environment overall. Connections between animal welfare and human welfare are documented in various contexts; for example, animal abuse has been linked to higher rates of family and social violence [29], while programs that match dogs with prisoners can help foster positive emotions [30] and reduce reoffending [31]. Additionally, links between the environment and human welfare are well supported, with studies demonstrating that exposure to nature and green spaces increases human happiness [32] and health [33]. In the context of wildlife provisioning, particularly during an emergency, there is likely a suite of complex interactions between humans, animals, and the environment that must be navigated if provisioning advice is to achieve optimal welfare outcomes for all parties. That said, all parties cannot be expected to benefit optimally at the same time from the same resources.

It would be easy to assume that wildlife provisioning in general has obvious and immediate positive impacts on animal welfare through increased access to resources. However, this is highly likely to be context dependent and related to a range of variables such as provisioning intentions (e.g., for conservation, tourism, or recreation) and the appropriateness and execution of methods (e.g., food suitability, hygiene, and distribution of resources). Provisioning outcomes for animals can be complex and conflicting since the provisioning may increase the growth, survival, and reproductive output of individuals [15], but can also increase disease transmission [20], competition [34], predation [35], and aggression [36], leading to poor health outcomes for animals under some circumstances. In the context of provisioning in response to large-scale habitat destruction such as bushfires, little is known about the effect of provisioned resources on animal welfare overall. We might hypothesise that access to provisioned resources in the short term would alleviate hunger and thirst and increase the probability of survival for affected animals. However, these outcomes must be considered in conjunction with possible negative effects on animal health, behaviour, and interactions. 

The impact of wildlife provisioning on the environment is often overlooked in provisioning studies, with experimental outcomes frequently focused on only individuals or target species. However, some evidence suggests that provisioning can be detrimental to ecosystems by altering natural food webs [37], changing ecosystem processes such as seed dispersal [38], and encouraging the proliferation of non-native species [39]. Alternatively, under conservation settings such as following a catastrophic bushfire, provisioning might assist in sustaining natural wildlife populations or reducing habitat damage and protecting valuable natural areas (e.g., by reducing grazing pressure on regenerating areas).

Wildlife provisioning may compromise human welfare by affecting the mental health and welfare of provisioners. In the context of recreation, provisioning via backyard bird feeding has been shown to increase the connectedness of people with nature and increase feelings of relaxation [40]. Although supplementary feeding of wild birds can be technically difficult to accomplish, it is possible without deleterious effects on the health and welfare of bird populations [41]. Provisioning in response to an environmental disaster, however, is likely more complex and requires investigation. While exposure to decimated landscapes and starving animals is likely detrimental to the mental health of onlookers [42], the action of provisioning animals may decrease feelings of helplessness or hopelessness by allowing people to feel a sense of control during a crisis [43]. 

**Figure 2:**
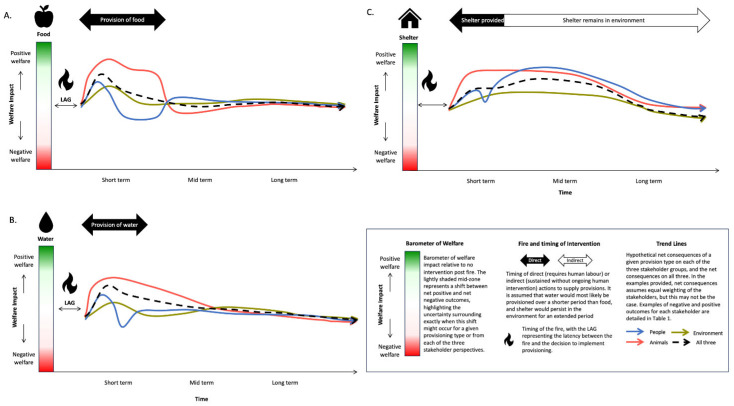
A conceptual illustration of some indicative consequences for each of the One Welfare stakeholder groups: animals, humans, and the environment in the short-, mid-, and long-term hypothetical best-case scenarios when provisioning wildlife with: (**A**) food, (**B**) water, and (**C**) shelter.

To develop wildlife provisioning advice that maximises positive welfare outcomes for humans, animals, and the environment, it is necessary not only to investigate the impacts of provisioning on each of these three elements, but also to consider the complex interactions amongst them and how these might change over time. While particular actions might achieve positive outcomes for one element of this triad, it may incur costs for another. For example, an action may make people feel good in the short term but compromise long-term animal health. We provide a visual conceptualisation of the ways that shelter, water, or food provisions might impact humans, some animals, and the environment over time (Figure 2). This concept map only demonstrates the way in which each element of the One Welfare approach must be considered in conjunction with the others, if optimal welfare outcomes are to be achieved. It focuses on best-case scenarios and is not intended to forecast outcomes for all species. Section 3 of the current article provides an experimental framework for addressing this knowledge gap.

The conceptual graphs (Figure 2) are based on a series of best-case assumptions that merit discussion. The initial lag is the latency between fire and the decision to implement provisioning and/or access the fire ground. For the purposes of this commentary, we have assumed a longer lag time for provision of shelter than provision of food or water. Our representations of the three interventions are based on the assumption that shelter requires short-term investment in human time and capital but lasts longer term without additional human effort being required. For water, we have assumed that it may not be needed for as long as food, assuming rain occurs within the short-medium term. Meanwhile, when considering food, longer-term intervention may be required post-bushfire as it takes longer for the ecosystem to recover (i.e., for adequate natural forage to become available).

The conceptual graphs indicate the theoretical overall net benefits for all stakeholders (Figure 2), considering some of the potential positive and negative welfare consequences for each stakeholder group (Table 1). These lines on the graphs depict the notional approximated middle ground among the three stakeholders, but we acknowledge that there may be instances where more or less weighting may be given to one or other stakeholders when deciding about the net benefits of an action. For example, if an intervention is expected to be too costly to implement, or may pose too much risk to personnel, then human outcomes may be given a greater weighting.

The detailed assumptions relating to animals, people, and the environment that underpin each of the three conceptual graphs can be considered separately, as follows.

### 2.1. Food

For animals, we have assumed that they will find the food and consume it quite quickly with benefits being critical if there is no other food in the environment. We note that faecal contamination could reduce the net benefits if parasite and disease transmission is amplified when the food provisions are frequented by unnaturally high concentrations of animals. Benefit declines rapidly once the food is removed if the natural vegetation has not recovered sufficiently. For people, we assume that the likely self-validation benefits of providing food may wane significantly over the course of intervention as regularly replenishing the resource can be physically demanding and costly. This may be partially countered by the positive perception that animals are benefitting from food provisioning, which is more obvious if there is clear evidence of consumption. This may contrast with the monitoring of water where observed levels of water in an open container do not permit differentiation between evaporation and animal consumption. Lastly, for the environment, we envision initial positive benefits from allowing animals to persist in the local environment, but caution that animals congregating around any focal resource may lead to trampling and soil compaction around feeding stations, along with faecal contamination.

### 2.2. Water

For animals, we have assumed that the water will be found and used by animals quite quickly, so they benefit from having ready access to uncontaminated water (and do not need to move further afield to find water). We anticipate that these benefits may not be as great as those from food if subsequent rainfall provides an alternative water source. Benefit declines once the water is removed, but natural rainfall will eventually replace the provisioned water (unless drought prevails). Here, the assumption is that, if well-maintained (i.e., regularly maintained when refilled), the water does not become contaminated to the extent that it facilitates disease transmission. However, this maintenance task comes at a human cost, i.e., can be very labour-intensive to cart water into remote locations. Clearly, faecal contamination of provisioned water has the capacity to reduce the net benefits if parasite and disease transmission is amplified. For people, we again anticipate the rewards of self-validation in the short term but acknowledge that this may wane significantly over the course of intervention as regularly replenishing water can be physically demanding. Lastly, for the environment, we again envision initial positive benefits from allowing animals to persist in the local environment, but note that this may be countered by the negative effects of anthropogenic congregations of animals, along with faecal contamination.

### 2.3. Shelter

Finally, we consider the consequences of providing shelter for animals. There is expected to be a delay before animals find the shelter and use it. Once they have found it, there is a net benefit in the short to mid term but this effect wanes over the long term if the shelter is not maintained (e.g., shelters degrade over time or may become used by non-target or invasive species). The consequences of shelter on people reflect the effort and satisfaction that may arise when shelter is provided. Self-validation is expected to wane a little in the short term due to the effort and resources required to build and install shelter. However, the validation factor is likely to endure for some time, especially if people are monitoring the use of the shelter in some way and obtaining an additional benefit of positive interaction with animals. This benefit is expected to last as long as there is a sense of achievement. Finally, and again based on best-case scenarios, the consequences of shelter on the environment are likely to be positive in providing structural complexity, shade, and refuge for wildlife so that they persist in the local ecosystem. However, net benefit decreases over time as ‘natural’ shelter returns for some species but this eventuality depends on the type of species-specific shelter requirements and how readily natural shelter recovers—an outcome that may arise more rapidly for species dependent on ground vegetation for shelter than for hollow-dependent species.

We propose that the costs and benefits of wildlife provisioning to each stakeholder group could be assessed through a range of health and welfare outcomes as outlined in Table 1. These outcomes could be assessed through the framework outlined below in Section 3.

## 3. Approaching the Problem

If recommendations are to be made to guide future wildlife provisioning responses to environmental disasters, we must assess the costs and benefits of wildlife provisioning practices to animals, humans, and the environment over various time frames. To this end, we must also understand what is already known about wildlife provisioning (particularly in the context of replacement resources in a conservation setting), how provisioning sits within relevant legislation and policies, and the views, attitudes, and experiences of stakeholders.

This leads us to propose a five-step approach examining the: 1. existing literature; 2. local legislation; 3. views of stakeholders; 4. emerging data; and 5. modelling from fire events. These steps are explored below.

### 3.1. Review of the Existing Literature

There is a clear need for a systematic meta-analysis of the international peer-reviewed and Australian lay literature relating to wildlife provisioning. This review should focus on studies where food or water were intentionally provided to animals in the wild. Relevant works should be identified using keyword searches on major databases, by examining the reference lists of published papers, and through direct contact with partner and supporting organisations. They should then be reviewed for relevance, then coded for species traits and scored for attributes likely to affect the outcomes of wildlife provisioning, including target species’ life-history traits, habitat, type of provisioning, method of provisioning, and reason for provisioning. Measures of success from each recorded intervention should be coded according to the scale of the success criterion, e.g., at individual animal, species, or ecosystem levels. This process should facilitate synthesis of current knowledge and, importantly, identify gaps to be investigated, especially as they relate to the context of environmental disasters.

### 3.2. The Local Legislation

In parallel, we see the need for a review of current advice, policy, regulations, and legislation relating to wildlife provisioning in each state/territory jurisdiction, from government and non-government agencies and organisations. The Australasian Legal Information Institute (AustLII) database [44] collates state and territory biodiversity acts, regulations, and government agency policies. A comprehensive review of relevant documents will determine the current legal status of provisioning activities on public and private land. Alongside legal frameworks, it is also critical to document the policies of major national private land conservation and state-based wildlife rehabilitation organisations. 

Given that some aspects of provisioning are illegal in some jurisdictions, for example, the feeding of any native wildlife in Western Australia (Section 155 of the Biodiversity Conservation Act 2016 (WA)), it is critical that any wildlife provisioning recommendations align with government policy, regulations, and legislation relating to provisioning in each state/territory jurisdiction. 

Based on this desktop exercise, in-depth interviews should be conducted with key staff from state and territory government agencies, conservation organisations, wildlife rehabilitators, and animal welfare organisations to gather detailed information on the drivers of current policies and track any changes in policy since September 2019.

### 3.3. Understanding the Views of Stakeholders

The relevant stakeholders in any discussion of wildlife provisioning should include wildlife rehabilitators, animal welfare groups, Traditional Owners, private land conservation groups, government agencies, and community groups. A national stakeholder survey should investigate stakeholders’ provisioning actions during the bushfires of 2019–2020, as well as their motivations, knowledge, experiences, and values. Demographic data (gender, age, ethnicity, education, location) across populations of stakeholders will permit selection of a targeted representative cohort of individuals to enable a longitudinal study and analysis of the relationships among respondents’ actions, knowledge, and motivations.

Interviews with representatives from key stakeholder groups with a direct interest in wildlife provisioning should also be undertaken (drawn from government, conservation, wildlife rehabilitation, and the community) to permit a deeper understanding of individual motivations, ideas, and plans for future provisioning. It is worth identifying any differences in the motivations that characterize provisioners and regulators; for example, recent studies have found that wildlife rehabilitators view their work as having both animal welfare and conservation aims [45]. This will inform what motivating factors are more likely to influence stakeholders’ uptake of advice. An understanding of the experiences of provisioners will also aid in assessing the relationship between provisioning activities and human welfare during an environmental disaster.

This approach to data collection will inform a review of wildlife provisioning activities in 2019/20 or thereafter. Collated data may become accessible on the location, target species, period of provisioning, quantities provided, wildlife presence, and use of provisioned resources, and other useful outcomes. This information may emerge from both direct responses to survey questions, and from alternate sources provided by survey respondents (for example, organisational reports, raw camera trap data, or internal databases). It has the potential to provide critical insight into the most effective strategies for the feeding of various animals, including location and placement of food, water, and shelter stations, types of feeders/drinkers/shelters, as well as appropriate species-specific feed items and shelters, if measures of success are provided. At this stage, theorised best practice would reflect the findings of the preceding steps, as well as advice from stakeholders.

### 3.4. Emergent Data

One of the specific aims of the proposed approach should be to use data from activities conducted in recent and emerging bushfire seasons, as well as surveys and consultation with experts across a range of disciplines (animal welfare, nutrition, environmental science, animal behaviour, wildlife medicine and pathology, and bioethics) to evaluate elements of provisioning risk and propose strategies to mitigate against them. Analysis of data collected via survey respondents, interviewees, and through the recently launched Wildlife Assist project, designed to collect targeted provisioning data [46], from past and future bushfire seasons will feed into this risk assessment and mitigation process and inform the future guidelines for supplementary food and water during times of disasters for improved outcomes for all native species. 

The Wildlife Assist project [46] enables stakeholders to access provisioning advice on the website and record detailed GPS-linked information on supplementary provisioning interventions through the app or website. A key functionality of the project is that it allows groups to collaboratively install, maintain, and monitor the assistance they are providing to wildlife. The information generated aims to provide a comprehensive database of provisioning information in future emergencies. The project also provides an opportunity to communicate with users via direct messages regarding the latest advice, the status of on-ground actions, and provide connectivity between the various stakeholders: landholders, wildlife rehabilitators, government, and wildlife and conservation organisations.

### 3.5. Field Studies and Modeling from Fire Events

Finally, we propose that field studies be undertaken to experimentally assess wildlife provisioning in association with bushfire. There is merit in sourcing data on low and high intensity fires. Specifically, hazard reduction fires, which are planned months in advance, provide an experimental framework where before–after control–impact (BACI) monitoring can be established, whereas bushfire events provide opportunities for post-fire (after) control–impact experiments. BACI designs more accurately estimate the true effects of environmental impacts [47], but have the disadvantage that they can only be planned around hazard reduction fires, which are of lower intensity than uncontrolled bushfires and therefore may not reflect real-world outcomes. 

Such studies should be specifically designed to assess the potential for positive and negative outcomes of provisioning at the individual, population, and ecosystem levels. Interventional studies should use passive monitoring methods, such as motion-triggered remote cameras, to record the usage of resources by species and to help assess any changes in species diversity and abundance, predator–prey interactions, and animal behaviour. This is a very important consideration since changes in the structure and functioning of species assemblages can have multiple consequences at the community and ecosystem levels [48]. Adding acoustic detectors may assist with the identification of species [49] that are present but are not identifiable on camera, e.g., they may not be using the provisioned resource but are hunting species that are. By assessing the use of provisions by different species, modelling can reveal the suitability of resources at various points in time after the fire and the extent to which provisioning is likely to confer positive health and welfare benefits for individuals and populations of different species. Through quantifying the behavioural interactions within and between species at provision points, one can determine the likelihood that wildlife provisioning increases the potential for negative welfare in the form of increased stress levels and/or increased capacity for disease transmission through increased intra- and hetero-specific contact rates and/or contact with contaminated substrate. Through measuring the effects of wildlife provisioning on species richness and diversity, one can assess the extent to which wildlife provisioning supports population recovery post-fire. This information is critical for a thorough assessment and cost–benefit analysis of wildlife provisioning after catastrophic fires and has the potential to strongly influence public policy in this area.

#### 3.5.1. Hazard Reduction Fires

Hazard reduction fires, although not as hot or vast as extreme bushfires, provide a unique opportunity to collect data on wildlife both before and after a fire due to their predictable nature—a critical attribute that is lacking when studying wildlife provisioning in response to an unpredictable bushfire. This type of assessment allows for conclusions to be drawn about not only the effects of provisioning on wildlife, but also the effects of both provisioning and fire, as well as their interactions. This approach would also give the power to address a criticism of wildlife provisioning—that it may unintentionally support the invasion of exotic predators into the system, thereby compromising individual welfare and population persistence.

**Figure 3:**
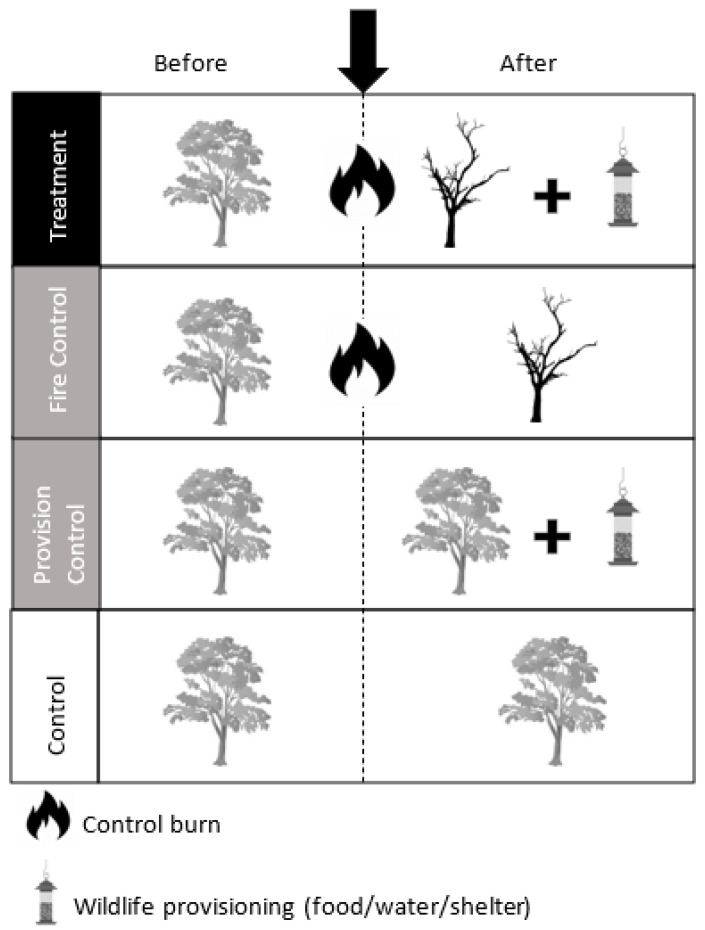
Proposed experimental approach to assess wildlife response to provisioning, utilizing a BACI (Before/After Control/Intervention) design in association with a hazard reduction (control) burn. Each of the four treatment and control conditions depicted would be replicated at multiple sites.

To test for the effects of fire and wildlife provisioning independently, a suggested experimental design is a before–after control–impact (BACI) study [47]. This design would allow multiple replicates of the experimental design across unburnt and burnt habitat (see Figure 3): Control: no provision before or after and no fire; Provisioning Control: provisioning in unburnt habit to control for the impacts of provisioning in the absence of fire; Fire Control: no provisioning before or after fire, testing for changes in the ecosystem as a result of fire irrespective of provisioning occurring; and Treatment: provisioning after the fire. At each control or treatment site, the use of provisions and behaviour at provision stations would be measured using motion-activated video cameras, and species diversity and abundance would be assessed using a standard camera trap array [50] in place before and after fire/provisioning.

#### 3.5.2. Future Fires

Collaborative work with supporting organisations engaged in wildlife provisioning (e.g., government department of national parks, non-government conservation land managers, wildlife rehabilitation and animal welfare organisations) is needed to collect standardized wildlife provisioning data, e.g., via the Wildlife Assist project or similar, when provisioning is deployed in response to bushfire events. Required data include the amount and type of resources provided, location, wildlife targeted, and use of resources by animals, among other outcomes. If the opportunity arises, wildlife provisioning trials should also be conducted and monitored in the ‘after’ experimental design (see Figure 3). Through these experiments, and in collaboration with stakeholders, emergent data will inform the development of best practice guidelines for wildlife provisioning.

## 4. Synthesizing the Information

Synthesizing the stakeholder survey, literature review, and experimental data can inform best practice guidelines for wildlife provisioning that are responsive to emergent data and ongoing monitoring. Such guidelines must be feasible to implement, minimize identified risks, and optimise welfare outcomes for affected wildlife, humans, and the environment. The provisioning response to the 2019–2020 fires suggests that there has never been a better time to influence policy in relation to wildlife welfare in this context. Stakeholder engagement strategy should focus on influencing policy change, guiding on-ground actions, and identifying knowledge gaps requiring further research.

It is likely that developing and reviewing a single national set of best practice guidelines for wildlife provisioning across Australia would be problematic due to differences in state legislation and the range of conditions under which provisioning might or might not be implemented. Metrics that are likely to vary include the scale of damage to the environment, the extent of human intervention required, and the selection of habitats, landscapes, and species assemblages that might benefit. However, evidence collected through the research proposed here will assist in providing a starting point for best practice, with more in-depth studies to follow and continuous improvement of knowledge and guidelines. Importantly, the evidence generated can be used to inform future decision making in an emergency—a prospect that was previously lacking. Additionally, increased collaboration among entities committed to the welfare of animals will ensure clearer and more effective messaging for interested parties and the public. These outcomes have obvious benefits for animal welfare by ensuring that, when human intervention occurs, it is undertaken in a strategic and consistent manner that balances the short-, mid-, and long-term welfare needs of animals, humans, and their environment.

It should be noted that the findings of the proposed research may indicate that the negative impacts of wildlife provisioning in an emergency outweigh the benefits, in which case any evidence-based recommendations might not align with community and stakeholder expectations. Under such circumstances, it would be important to explore alternative options for human intervention and action in an emergency that would satisfy the human need to assist while minimising any negative impacts on the welfare of animals and the environment. Lastly, a key aim of wildlife provisioning is to support wildlife remaining in the wild; there is significant merit in attempting to decrease the number of animals taken into veterinary or voluntary wildlife rehabilitation. Critically, parallel research is required to understand the outcomes of animals taken in for treatment and their survival rates following release back into the wild.

## 5. Conclusions

There is strong evidence indicating that future bushfire seasons will become longer and more intense in Australia and elsewhere, putting the welfare and survival of millions of wild animals at risk every year [25]. Understanding the impacts of supplementary resource interventions and contributing to the development of best practice information is crucial to inform the response to the next major fire event.

Engaging wildlife provisioners and the broader conservation community will encourage beneficial interventions, reduce the risk of negative impacts, and build an evidence base for future wildlife provisioning activities. If this approach were implemented, it is anticipated that best practice recommendations for stakeholders in different contexts would emerge. Beyond the proposed experiments, ongoing data collection using emergent technology such as the Wildlife Assist app and longitudinal analysis will address shifting research priorities as the climate changes. The ordered collection of the necessary evidence relevant to each of the three stakeholder groups in the One Welfare framework has the greatest potential to support an informed policy platform on wildlife provisioning across Australia that is feasible, legal, and sustainable. We recognise that there are potential conflicts or challenges in their implementation but, without the proposed evidence base and the One Welfare framework, we risk a repeat of the ad hoc approach to provisioning prevalent in the Black Summer bushfires and we will remain in the dark as to whether these activities are more beneficial than harmful.

## Figures and Tables

**Table 1:** Health and welfare outcomes that may reflect the measurable effects of wildlife provisioning post-fire.

Timeframe	Stakeholder Group					
	Animals		Humans		Immediate Environment	
	+ve	−ve	+ve	−ve	+ve	−ve
Short term	Hydration and sustenance	Abnormal intra- and inter-specific social interactions	Self-validation	Feelings of helplessness		
	Physical and thermal comfort	Exposure to predators	Positive interaction with animals	Stress and financial burden	Reduced grazing of emergent plants	Trampling emergent plants
Mid term	Shelter from predation	Unhealthy body condition	Building a sense of community	Exhaustion/ burnout	Maintaining populations and associated ecosystem services	Faecal and parasitic contamination
		Unhealthy bodyweight		Grief		Erosion from footfalls
		Parasite burden		Zoonotic disease		Emissions from carting provisions
Long term	Altered population density	Altered population density	Increased inclination to provision or support conservation in future	Guilt		Compromised soil health
		Dependence on extraneous resources	Sense of achievement	Trauma reactions		Shifts in biodiversity
				Compromised mental health/ resilience	Sustained biodiversity	Invasive fauna and flora

## Data Availability

Data are contained within the article.
